# Signalling by CGRP and Adrenomedullin in the Cerebellum and Other Systems

**DOI:** 10.1100/tsw.2001.434

**Published:** 2001-02-23

**Authors:** David Poyner, Heather Cater, Nick Hartell, Alex Conner, Debbie Hay, Steve Howitt, Dave Smith

**Affiliations:** ^1^ Pharmaceutical Sciences Research Institute, Aston University, Birmingham, UK; ^2^ Department of Biosciences, University of Birmingham, Birmingham, UK

## Abstract

The best characterised signalling pathway activated by both CGRP and adrenomedullin is stimulation of adenylate cyclase via Gs. However, it is clear that in some circumstances the peptides can activate other signal transduction pathways, e.g., increases in intracellular calcium. Many of these signalling pathways can be observed in cultured cells but it is important also to examine isolated tissues to discover the full repertoire of transduction events. In the rat cerebellum there are receptors that respond to both CGRP and adrenomedullin. These seem to be located postsynaptically on Parallel Fibre nerve terminals and modulate transmission to Purkinje cells. Adrenomedullin acts via cAMP, apparently to augment neurotransmitter release. By contrast, CGRP decreases transmitter release, via a non-cAMP mediated pathway. We are currently examining the role of NO and tyrosine kinases in the responses to these peptides.

